# Fungal vaccines: so needed, so feasible, and yet so far off

**DOI:** 10.1172/JCI199451

**Published:** 2025-11-17

**Authors:** Arturo Casadevall

**Affiliations:** Department of Molecular Microbiology and Immunology, Johns Hopkins School of Public Health, Baltimore, Maryland, USA.

## Abstract

Invasive fungal infections carry high morbidity and mortality, but there are no fungal vaccines. In this issue of the *JCI*, Okaa et al. report that endonuclease 2 (Eng2), an antigen shared by the *Blastomyces*, *Histoplasma*, and *Coccidioides* species of fungi, elicits protective immunity in mice against blastomycosis, histoplasmosis, and coccidioidomycosis. These results establish a common antigen that can elicit protection against multiple mycoses, encouraging the development of a pan-fungal vaccine. The road to fungal vaccines is made difficult by the need for effectiveness in immunocompromised individuals, the sporadic nature of fungal disease, and the economics of vaccine development. Despite these hurdles, there is optimism that such vaccines can be developed and perhaps find usefulness as adjuncts to antifungal therapy.

Invasive fungal diseases (IFDs) are high-consequence, low-probability events with high morbidity and mortality. Today, fungal diseases are responsible for 3.8 million deaths worldwide (1), and their prevalence is increasing because of increased numbers of immunocompromised individuals and the ongoing HIV pandemic. In general, IFDs are difficult to diagnose and treat, with therapy usually requiring prolonged courses of antifungal drugs, and the outcome often depends on the underlying diseases and extent of immunosuppression. Unfortunately, there are no licensed vaccines to prevent any fungal disease (2).

In this issue of the *JCI*, Okaa et al. (3) report that endoglucanase 2 (Eng2), an enzyme antigen found in *Blastomyces, Coccidioides,* and *Histoplasma* spp., each of which is responsible for major human mycoses, elicited protective immunity in a human immune system humanized mouse model. Furthermore, CD4^+^ T cell responses to Eng2 were found in individuals who recovered from these mycoses, indicating recognition of this enzyme antigen by cell-mediated immunity during infection. This work has important implications for vaccine design, since Eng2 is a candidate antigen for antifungal vaccines, as well as diagnosis, because T cell memory can be exploited in diagnosis and epidemiological studies of fungal infection in endemic regions.

Eng2 is shared among the three fungal species, but sequence variation precludes cross-protection: immunization with the enzyme from one species did not protect against the other two species (Figure 1). However, immunization with the species-specific homologous Eng2 protein robustly protected mice when they were challenged with the respective fungal species. Although this finding seems to predict that a vaccine using one Eng2 protein cannot protect against all three mycoses, vaccine design could overcome this limitation by constructing a polyvalent preparation composed of Eng2 from each fungal species, similar to designs already in use to protect against such antigenically variable pathogens as *Streptococcus pneumonia* and *Neisseria meningitidis*. Hence, the results of this work are a considerable advance toward the design of a broad antifungal vaccine that would protect against these dangerous mycoses.

## Fungal vaccines and natural immunity form multilayered protection

Eng2 joins a long list of fungal antigens that have been shown to elicit protective immunity (4). Polysaccharides, enzymes, and cell wall proteins have each been shown to be effective experimental vaccines against several major pathogenic fungi. In fact, there is no shortage of antigens that can elicit protective immunity against fungi, and dozens of vaccine candidates have shown promise in animal models (4). One of the paradoxes in the field of medical mycology is that, although fungal diseases are difficult to treat, it is relatively easier to protect against them, at least in experimental animal models. Acquired immunity to fungal pathogens is multilayered, relying on both arms of the adaptive immune response (5), which provides redundancy in immune defenses. For example, it is possible to elicit protection against experimental cryptococcosis with vaccines that elicit only humoral (6) or cellular immunity (7).

The relative success of experimental vaccines against major pathogenic fungi contrasts with the difficulty in making effective vaccines against other types of pathogenic microbes such as HIV, HSV-1 and -2, *Mycobacterium tuberculosis*, and *Plasmodium falciparum*. Although the topic of virulence versus success in generating a vaccine has not been explored, it is striking that for the example pathogenic microbes listed above — which cause disease in immunologically intact hosts — vaccine development has been difficult; in contrast, for most pathogenic fungi, which cause invasive and complicated disease primarily in immunocompromised hosts, there are more promising vaccine candidates (4). In this regard, it may be useful to think about vaccines as adding a layer of protection over that existing in natural immunity. Among immunocompetent individuals, not all infections with highly pathogenic microbes progress to disease, which indicates that even immunologically naive hosts have formidable antimicrobial defenses. Hence, it may be that for microbes capable of defeating intact immunity, the protection added by vaccines is incrementally smaller than for microbes that cause invasive disease primarily in immunocompromised hosts, such as the pathogenic fungi.

## Immunocompromised individuals stand to benefit from fungal vaccines

Humans are remarkably resistant to invasive fungal diseases, which is believed to be the result of high mammalian body temperatures that create a thermal exclusionary zone for most fungal species, in addition to advanced immunity in the form of innate and adaptive immune mechanisms (8). Consequently, IFDs occur primarily in immunocompromised individuals, who now constitute 6.6% of the US population (9). The fact that IFDs occur primarily in immunocompromised individuals poses the additional challenge that any vaccine strategy to protect these vulnerable hosts must elicit protective immunity in the setting of impaired immunity. However, the experience with vaccines against varicella zoster virus, SARS-CoV-2, and other vaccines shows that these can elicit sufficient immunity in immunocompromised hosts to provide some protection and/or ameliorate the course of infection. For the pathogenic fungi that rarely cause disease in immunologically intact hosts, the amount of immunological boosting provided by vaccines in immunocompromised hosts may be sufficient to stave off disease.

Assuming that antifungal vaccines can be made and that they elicit immune responses in immunocompromised hosts, the next hurdle will be establishing efficacy in vulnerable populations. The fact that most immunocompromised individuals do not develop IFDs means that vaccines are meant to prevent a high-consequence but low-probability event, and the latter implies the need to conduct targeted clinical trials to establish efficacy. Such trials are feasible since the epidemiology of IFDs in immunocompromised individuals is known, and thus it may be possible to target cohorts at greatest risk. However, such trials will be costly, and the results will be complicated by participant heterogeneity and the use of prophylactic antifungal drugs in vulnerable individuals.

Vaccine economics pose a major challenge to the development of vaccines against IFDs because vaccine development is costly. Moreover, the fact that fungal diseases are a problem primarily for immunocompromised individuals means that the size of the market is smaller than for vaccines developed for the general population. Nevertheless, because IFDs are high-consequence events with high morbidity and mortality, a vaccine may prove to be commercially successful even for niche populations. In this regard, we can expect a vaccine that prevents aspergillosis or coccidiomycosis in organ transplant recipients or pregnant women, respectively, would find use in those at risk. mRNA vaccine technology could substantially lower costs for vaccines based on protein antigens, and the Eng2 protein described by Okaa et al. (3) would be suitable for development as an mRNA vaccine.

## Barriers to the development of fungal vaccines

While the development of preventive vaccines is hindered by the sporadic nature of IFDs in vulnerable individuals, developing therapeutic vaccines for fungal diseases could find an easier road. In this context, IFDs tend to be chronic, with prolonged courses, which would allow time for vaccination to elicit an immune response that aids antifungal therapy in clearing the infection. While most currently available vaccines are preventative, it is noteworthy that both the rabies and hepatitis A vaccines are effective after infection has taken hold in an individual. Hence, the principle for therapeutic vaccines already exists, and individuals who are at high risk for considerable morbidity and death represent a targeted population for testing fungal vaccines in which establishing therapeutic efficacy may be considerably easier than showing preventative efficacy. If therapeutic vaccines are first shown to be safe and effective in therapy, that could ease the regulatory road to finding preventative use in vulnerable populations.

Despite these hurdles, some vaccines against fungi have reached clinical development. A vaccine composed of formaldehyde-killed spherules of *Coccidioides immitis* was tested in a phase III trial in the early 1980s. The results showed a slight reduction in cases among vaccinated individuals that did not reach statistical significance (10), although the study may have been underpowered. In the late 1980s, an oral vaccine for the prevention of vaginal candidiasis composed of ribosomes plus membrane proteoglycan from a nonencapsulated *Klebsiella pneumoniae* as an adjuvant was effective in reducing recurrent disease in a phase II trial (11). More recently, a vaccine against *Candida albicans* using the N-terminal portion of the agglutinin-like sequence 3 (Als3) was tested in women for the prevention of recurrent vaginal candidiasis and was found to reduce the likelihood of recurrences (12). It is noteworthy that the Als3 vaccine for the prevention of recurrent candidiasis was a therapeutic vaccine, since it did not prevent infection but rather prevented disease (13). Although the Als3 vaccine still has a significant developmental road ahead until possible licensure, its success in early trials provides strong encouragement for the promise of antifungal vaccines.

In summary, while there is great need for the development of vaccines to prevent IFD in vulnerable populations and the experience with experimental fungal vaccines in animal models and limited human experience shows that these vaccines are feasible, none is expected in the near horizon. Nevertheless, the report by Okaa et al. (3) showing that a single-enzyme antigen can provide protection against three of the major mycoses is an important contribution because it potentially simplifies the development of an effective pan-fungal vaccine (3). The history of successful vaccines, from Jenner in the late 1700s to the development of mRNA vaccines pioneered by Katalin Kariko and Drew Weissman (14), is the story of committed individuals who often struggled against great odds and skepticism to prevail in finding, testing, and developing such immunogens and, in doing so, brought great benefit to humanity. The fungal field is fortunate to include many such scientists and physicians who are currently struggling with the challenges described above, but the history of vaccinology provides great confidence that they will succeed.

## Funding support

This work is the result of NIH funding, in whole or in part, and is subject to the NIH Public Access Policy. Through acceptance of this federal funding, the NIH has been given a right to make the work publicly available in PubMed Central.

- NIH awards R01AI162381, R01AI152078, and R01AI052733 (to AC).

## Figures and Tables

**Figure 1:**
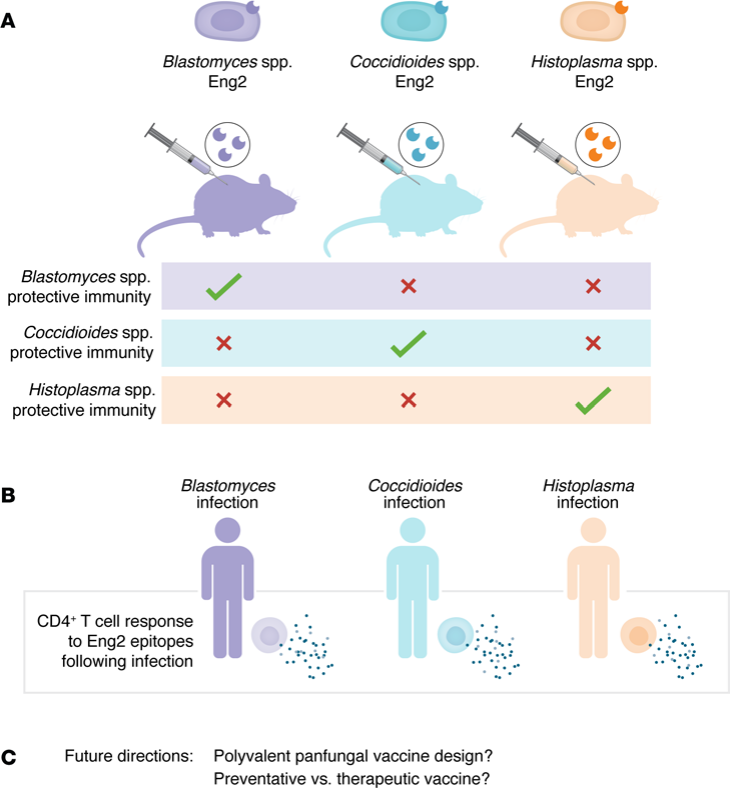
Eng2 elicits protective immunity against multiple pathogenic fungi. (**A**) Using a human immune system humanized mouse model, Okaa et al. (3) determined that immunization with the fungal enzyme antigen Eng2 protected against infection by *Blastomyces*, *Coccidioides*, or *Histoplasma* spp., representing three major human mycoses. However, sequence variation in Eng2 prevented cross-species protection. (**B**) Okaa et al. also identified CD4^+^ T cell responses to Eng2 in patients who had recently recovered from these three mycoses, supporting the interpretation that recognition of Eng2 can elicit cell-mediated immunity during infection. (**C**) The findings encourage investigation of a trivalent vaccine strategy targeting Eng2, which could provide pan-fungal protection as a preventative or even therapeutic approach. A therapeutic strategy could particularly benefit immunocompromised individuals for whom fungal infection can pose major risks.

## Version 1 11/17/2025

Electronic publication
